# New Green Polymeric Composites Based on Hemp and Natural Rubber Processed by Electron Beam Irradiation

**DOI:** 10.1155/2014/684047

**Published:** 2014-01-28

**Authors:** Maria-Daniela Stelescu, Elena Manaila, Gabriela Craciun, Maria Dumitrascu

**Affiliations:** ^1^National Research and Development Institute for Textile and Leather-Leather and Footwear Research Institute, 93 Ion Minulescu Street, 031215 Bucharest, Romania; ^2^National Institute for Lasers, Plasma and Radiation Physics, Accelerators Laboratory, 409 Atomistilor Street, 077125 Magurele, Romania

## Abstract

A new polymeric composite based on natural rubber reinforced with hemp has been processed by electron beam irradiation and characterized by several methods. The mechanical characteristics: gel fraction, crosslink density, water uptake, swelling parameters, and FTIR of natural rubber/hemp fiber composites have been investigated as a function of the hemp content and absorbed dose. Physical and mechanical properties present a significant improvement as a result of adding hemp fibres in blends. Our experiments showed that the hemp fibers have a reinforcing effect on natural rubber similar to mineral fillers (chalk, carbon black, silica). The crosslinking rates of samples, measured using the Flory-Rehner equation, increase as a result of the amount of hemp in blends and the electron beam irradiation dose increasing. The swelling parameters of samples significantly depend on the amount of hemp in blends, because the latter have hydrophilic characteristics.

## 1. Introduction

Green composite combines plant fibers with natural resins to create natural composite materials. Natural fibers such as hemp, kenaf, flax, jute, sisal, and so forth have attracted interest, especially as a synthetic fibers substitute in the rubber and plastics industry. The advantages of natural fibers over synthetic are low cost, low density, acceptable specific strength properties, ease of separation, carbon dioxide sequestration, and biodegradability. In fibers-reinforced composites, the fibers serve as reinforcements by giving strength and stiffness to the composite structure [1, 2]. ı

In this paper the results of our study on obtaining and characterizating a new green polymeric composite based on hemp and natural rubber, crosslinked by irradiation with electron beam (EB) are presented. There are few studies regarding the use of hemp fiber for the achievement of composites based on natural rubber. Osabohien and Egboh [3] conducted a study on the use of bowstring hemp fiber as filler in natural rubber compounds, compared with carbon black. The hemp fibers/rubber had lower tensile strength (only 2/3 of the carbon black/rubber), but the hemp fiber/rubber showed superior hardness (1.26 times that of carbon black/rubber). So, hemp fibers could replace active fillers such as carbon black or silica from natural rubber. Silica is known to have adverse effects on health: silicosis, cancer (Group 1 according to IARC—the International Agency for Research on Cancer), autoimmune diseases, tuberculosis, kidney disease, and so forth, and in 1995, the IARC rated carbon black as IARC classification 2B—possibly carcinogenic to humans and definitely carcinogenic to animals [4–8]. Cho and collaborators [9] specify that an introduction of ecofriendly natural fibers to natural rubber can play a role not only in reinforcing rubber but also in reducing the amount of carbon black used in rubber and tire applications. In the mentioned study [3] the curing of natural rubber/hemp composites has been achieved by the classical method of crosslinking, that is, using sulfur and vulcanization accelerators. In another study [10], elastomer crosslinking was performed using benzoyl peroxide in order to obtain also a natural rubber/hemp composite. This method of crosslinking results in a better composite resistance to aging than those obtained by the classical method [11]. In addition to elastomer crosslinking, peroxide can perform chemical surface modification of hemp fibers [10]. As a result of our study, a green composite based on natural rubber and hemp was obtained and elastomer crosslinking was performed by EB irradiation, thus eliminating the use of sulfur and crosslinking agents which lead to the appearance of toxic substances (such as N-nitrosamines which are suspected in human carcinogens) [4, 12]. Crosslinking technology by EB irradiation is a relatively new technology globally. The first patented process for rubber curing by means of ionizing radiation was developed by Dunlop Rubber Co. Ltd. in 1956. Since that year, application of ionizing radiation in the polymer field has been investigated by many researchers who have developed modern, environmentally friendly, and fast techniques for polymer crosslinking and grafting [13–15]. Elastomers crosslinking using EB irradiation present a series of specific advantages over the traditional thermal curing, such as (1) lack of curing agents, except activators for rubber; (2) obtaining new highly pure materials (intended for medical devices, rubber items for food industry, toys for children, etc.); (3) enabling new rubber types which cannot be crosslinked chemically or can be hardly crosslinked by usual curing procedures to be processed into finished products with major industrial applications (aircraft, army, medicine); (4) the process is very fast and can be controlled precisely, it is *very clean*, requires *less energy*, permits greater processing speed, and operates at *ambient temperature*; (5) the electron beam can be steered very easily to meet the requirements of various geometrical shapes of the products to be cured; (6) the high penetrating power of radiation allows the efficient and uniform curing of thick polymeric articles; (7) the process is practically waste-free; (8) and no polymer degradation due to high temperature as EB crosslinking occurs at room temperature [13–18]. Because of their reliability, flexibility, low-cost, along with no environmental impact, the irradiation technologies are particularly attractive. The advantages of such technologies lead to the appearance of more and more EB irradiation stations: there are more than 1200 electron accelerators for industrial applications, intended particularly for polymerization, crosslinking, grafting, and so forth all over the world. Only in China, there are 45 industrial electron accelerators and 123 gamma radiation for various radiation processing applications. Malaysia has six EB accelerators for commercial use [13].

## 2. Material and Methods

### 2.1. Materials

In preparing the above polymer composites, the following materials were used: natural rubber (NR) Crep 1X (Mooney viscosity is 74 ML_1+4_ at 100°C, 0.32% volatile materials, 0.38% nitrogen, 0.22% ash, 0.021% impurities), antioxidant pentaerythritol tetrakis(3-(3,5-di-tert-butyl-4-hydroxyphenyl) propionate Irganox 1010, polyethylene glycol PEG 4000 (1.128 g/cm^3^ density, 4–8°C melting point range), and ground hemp (thread length of max 3 mm).

### 2.2. Sample Preparation

Blends were prepared on an electrically heated laboratory roller. For preparation of polymeric composites, the blend constituents were added in the following sequences and amounts: 100 parts natural rubber (NR) roll binding (2′), embedding 3 phr (parts to 100 parts rubber) PEG 4000 and 1 phr Irganox 1010 antioxidant (2′), adding 5, 10, 15, and 20 phr ground hemp (2–4′), and homogenisation of blends and removing from the roll in the form of sheet (4′). Process variables: temperature 25–50 ± 5°C, friction 1.1, and total blending time 8–14′. Plates required for physico-mechanical tests with sizes of 150 × 150 × 2 mm^3^ were obtained by pressing in a hydraulic press at 110 ± 5°C and 150 MPa.

### 2.3. Experimental Installations and Sample Irradiation

The samples were irradiated using the electron beam accelerator called ALIN 10 in the dose range of 15–60 Mrad. The ALIN 10 is a travelling-wave type, operating at a wavelength of 10 cm and having 164 W maximum output power. The accelerating structure is a disk-loaded tube operating in the *π*/2 mode. The optimum values of the EB peak current *I*_EB_ and EB energy *E*_EB_ to produce maximum output power *P*_EB_ for a fixed pulse duration *τ*_EB_ and repetition frequency *f*_EB_ are as follows: *E*_EB_ = 6.23 MeV, *I*_EB_ = 75 mA, and *P*_EB_ = 164 W (*f*_EB_ = 100 Hz, *τ*_EB_ = 3.5 *μ*s). The EB effects are related to the absorbed dose (*D*) expressed in Gray or J kg^−1^ and absorbed dose rate (D*) expressed in Gy s^−1^ or J kg^−1^ s^−1^ [19–21]. Layers of three sandwiched sheets covered in polyethylene foils were irradiated in atmospheric conditions and at room temperature of 25°C. Samples were irradiated with 75, 150, 300, and 600 kGy.

### 2.4. Laboratory Tests

#### 2.4.1. Mechanical Characteristics

*Tensile strength* tests were carried out with a Schopper strength tester with testing speed 460 mm/min, using dumb-bell shaped specimens according to ISO 37/2012. The tests measurement uncertainty was ±0.64 for tensile strength and ±2.95 for elongation at break. *Hardness* was measured by using a hardener tester according to ISO 7619-1/2011 using 6 mm thick samples (the tests measurement uncertainty was ±0.05). *Elasticity* was evaluated with a test machine of type Schob using 6 mm thick samples, according to ISO 4662/2009.

#### 2.4.2. Gel Content

The gel content was performed on crosslinked NR rubber (with and without hemp) to determine the mass fraction of insoluble NR (the network material resulting from network-forming crosslinking process) samples. The samples were weighed in the dry condition (*m*_*i*_) then immersed in the toluene during 3 days at room temperature in order to remove any scissioned fragments and unreacted materials. The samples were then dried in air for 6 days and in an oven at 80°C for 3 hours and reweighed (*m*_*s*_). The gel content was calculated as

(1) $$\begin{array}{c} \text{Ge}{\text{l}}_{\text{content}}=\frac{m_{s}}{m_{i}}\times 100, \end{array}$$

where *m*_*s*_ and *m*_*i*_ are the weight of the dried sample after immersion and the weight of the sample before immersion, respectively [22, 23].

#### 2.4.3. Crosslink Density

*The crosslink density* (*ν*) of the samples was determined on the basis of equilibrium solvent-swelling measurements (in toluene at 23–25°C) by application of the well-known modified Flory-Rehner equation for tetrafunctional networks. The samples (2 mm thick) were initially weighed (*m*_*i*_) and immersed in toluene for 72 h. The swollen samples were removed and cautiously dried to remove excess solvent before being weighed (*m*_*g*_) and, during this operation, the samples were covered to avoid toluene evaporation during weighing. Traces of solvent and other small molecules were then eliminated by drying in air for 6 days and in an oven at 80°C for 3 hours. Finally, the samples were weighed for the last time (*m*_*s*_), and volume fractions of polymer in the samples at equilibrium swelling *ν*_2*m*_ were determined from swelling ratio *G* as follows:

(2) $$\begin{array}{c} \nu_{2m}=\frac{1}{1+G}, \end{array}$$

where

(3) $$\begin{array}{c} G=\frac{m_{g}-m_{s}}{m_{s}}\times \frac{\rho_{e}}{\rho_{s}}, \end{array}$$

where  *ρ*_*e*_ and *ρ*_*s*_ are the densities of elastomer samples and solvent (0.866 g/cm^3^ for toluene), respectively.

The densities of elastomer samples were determined by the hydrostatic weighing method, according to the SR ISO 2781/2010 (the tests measurement uncertainty was ±0.09). By this method, the volume of a solid sample is determined by comparing the weight of the sample in air to the weight of the sample immersed in a liquid of known density. The volume of the sample is equal to the difference in the two weights divided by the density of the liquid. The samples crosslink densities, *ν*, were determined from measurements in a solvent, using the Flory-Rehner relationship:

(4) $$\begin{array}{c} \nu =-\frac{\text{Ln}\left(1-\nu_{2m}\right)+\nu_{2m}+\chi_{12}\nu_{2m}^{2}}{V_{1}\left(\nu_{2m}^{1/3}-\left(\nu_{2m}/2\right)\right)}, \end{array}$$

where *V*_1_ is the molar volume of solvent (106.5 cm^3^/mol for toluene), *ν*_2*m*_ is the volume fraction of polymer in the sample at equilibrium swelling, and *χ*_12_ is the Flory-Huggins polymer-solvent interaction term (the value of *χ*_12_ is 0.393 for natural rubber - toluene) [22, 23].

#### 2.4.4. Water Uptake Test

The effect of water absorption on fiber reinforced natural rubber composites is investigated in accordance with SR EN ISO 20344/2004. The samples were dried in an oven at 80°C for 2 hours and then are allowed to cool to room temperature in desiccators before weighing. Water absorption tests were conducted by immersing the samples in distilled water in bottles and kept at room temperature (23 ± 2°C). Samples were removed from the bottles at periodic intervals and the wet surfaces were quickly wiped using a clean dry cloth or tissue paper, and weights of the specimen after swelling were determined at regular intervals until no further increase in solvent uptake was detected. The moisture absorption was calculated by the weight difference. The percentage weight gain of the samples was measured at different time intervals. The water uptake was calculated as

(5) $$\begin{array}{c} \text{water}\;\;\text{uptake}\left(\%\right)=\frac{m_{s}-m_{i}}{m_{i}}\times 100, \end{array}$$

where *m*_*s*_ is the weight of the water saturated specimen at periodic intervals and *m*_*i*_ is the initial weight of the oven-dried specimen. The tests measurement uncertainty was ±0.04.

#### 2.4.5. Fourier Transform Infrared (FTIR) Spectroscopy

Changes in the chemical structure of natural rubber samples with/without hemp irradiated with 75, 150, 300, and 600 kGy were determined with an FTIR spectrophotometer—JASCO FT/IR 4200, by the ATR measurement method. Samples spectra are the average of 30 scans realized in absorption in the range of 4000–600 cm^−1^, with a resolution of 4 cm^−1^.

## 3. Results and Discussion

### 3.1. Mechanism of Crosslinking and Grafting of Polymeric Composites Based on Natural Rubber and Hemp by Electron Beam Irradiation

**Scheme 1:**
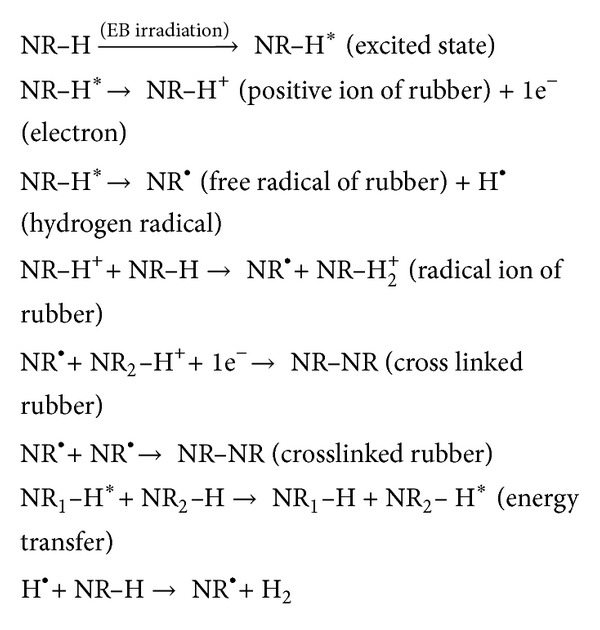
Mechanism for the radiation crosslinking of NR.

The effects of electron beam on polymers have been investigated by many researchers [24, 25] over the past few decades. Among the effects is that high energy irradiation causes crosslinking and degradation in polymers. These reactions are reported to follow the free radical mechanism. As a result of crosslinking, the tensile strength, elasticity, and modulus increase while the elongation at break decreases. Degradation, on the other hand, leads to a decrease in tensile strength, elasticity, and modulus [25]. Elastomer crosslinking by means of electron beam is done without heating and in the absence of vulcanization agents. One of the proposed mechanisms for the radiation crosslinking of NR is summarized in Scheme 1. Mechanisms for the radiation crosslinking of different rubbers were developed by the authors in other articles [14, 15, 26].

The chemistry of the process is based on macroradical formation from elastomer chains, which recombine, causing structuring.

Hemp fibers are obtained from the bast of the plant *Cannabis sativa* L. It grows easily—to a height of 4 m—without agrochemicals and captures large quantities of carbon. Long, strong, and durable, hemp fibers are about 70% cellulose and contain low levels of lignin (around 8–10%), hemicelluloses, lignin, waxes, and so forth. The fibers diameter ranges from 16 to 50 microns. Hemp fibers conducts heat, dye well, resist mildew, block ultraviolet light, and have natural antibacterial properties [27, 28].

**Scheme 2:**
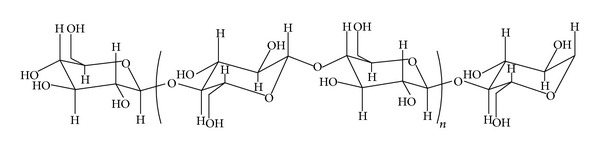
Structure of cellulose.

Cellulose chain consists of anhydro-*β*-dextroglucose, which are connected by *β*-glucosidic 1 → 4 bridges (see Scheme 2). The effects of electron beam irradiation on cellulose have been evaluated in several studies [29–32] and it was observed that the atmospheric oxygen affects the irradiated cellulose.

**Scheme 3:**
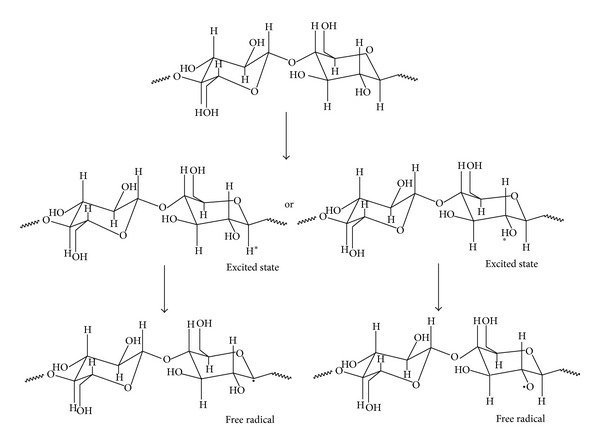
Radical formation on the cellulose chains.

**Scheme 4:**
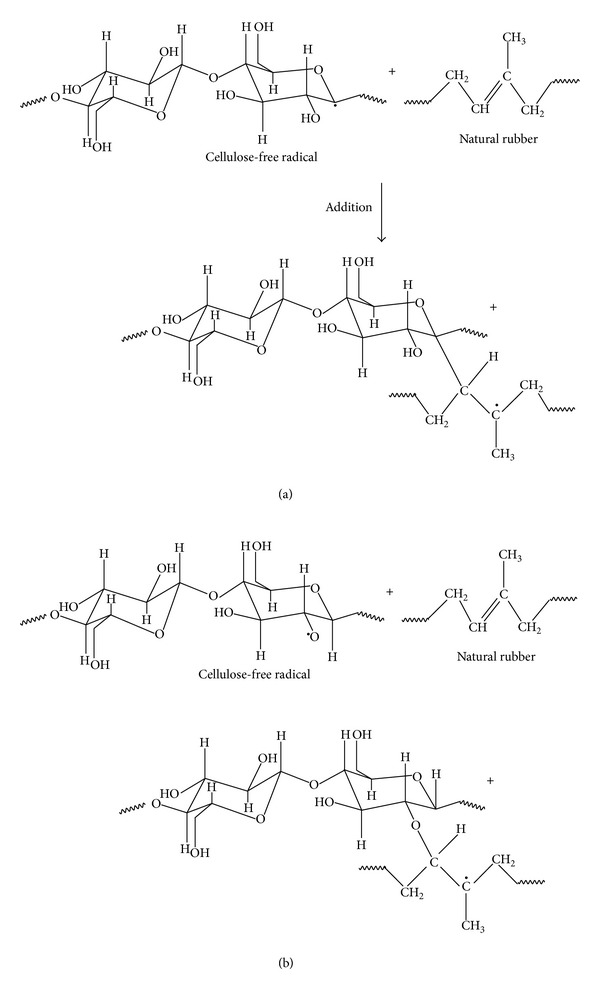
Proposed mechanism for the interaction between cellulose and natural rubber.

In our study, hemp fibers, having high cellulose content, are in the form of filler in a natural rubber matrix. So, atmospheric oxygen affects these types of fibers less than those irradiated in the mentioned studies [29–32]. By irradiation mainly occur crosslinking, grafting and degradation of these types of NR/hemp fibers composites. Crosslinking process leads to the increase of composites crosslinking degree and to the improvement of some physical and mechanical properties. NR macromolecules grafting leads to the formation of a grafted copolymer at the interface between the two phases which will significantly improve their compatibility leading to obtaining a polymeric composite having optimum properties.  Scheme 3 presents the formation of two macroradicals which can further react with NR macromolecules (Scheme 4) forming a grafted polymer at the interface. This grafted polymer can act as a material which can assure compatibility, improving adhesion between the two phases of NR and hemp mixture.

### 3.2. Physical and Mechanical Characteristics

**Figure 1:**
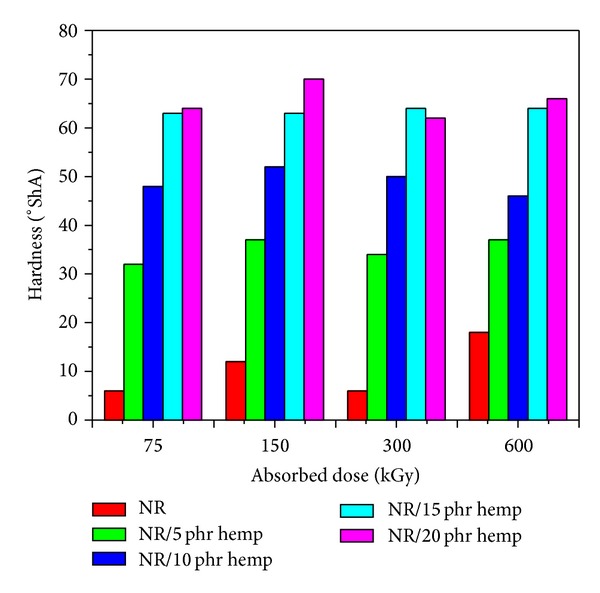
Hardness variation as a function of hemp amount and irradiation dose.

**Figure 2:**
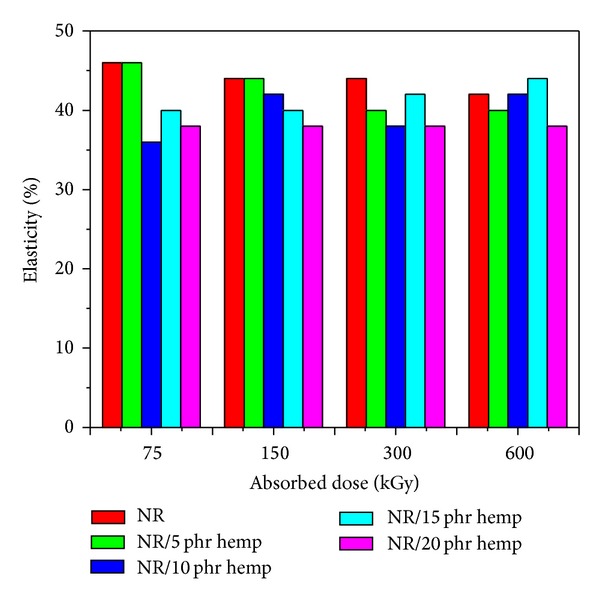
Elasticity variation as a function of hemp amount and irradiation dose.

**Figure 3:**
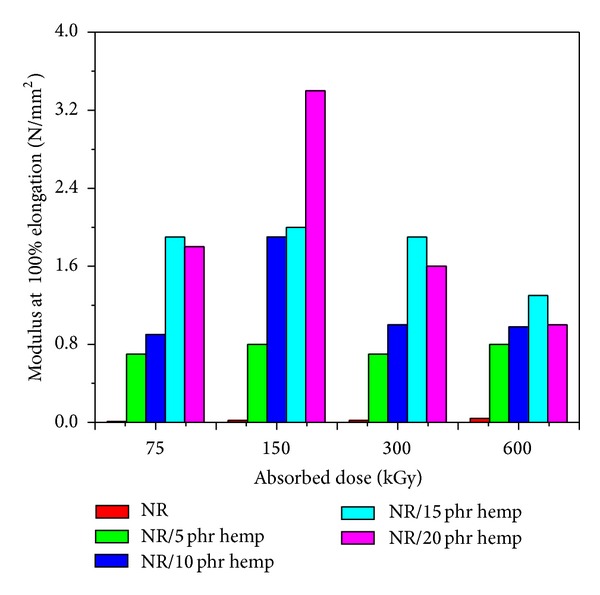
Modulus at 100% elongation variation as a function of hemp amount and irradiation dose.

**Figure 4:**
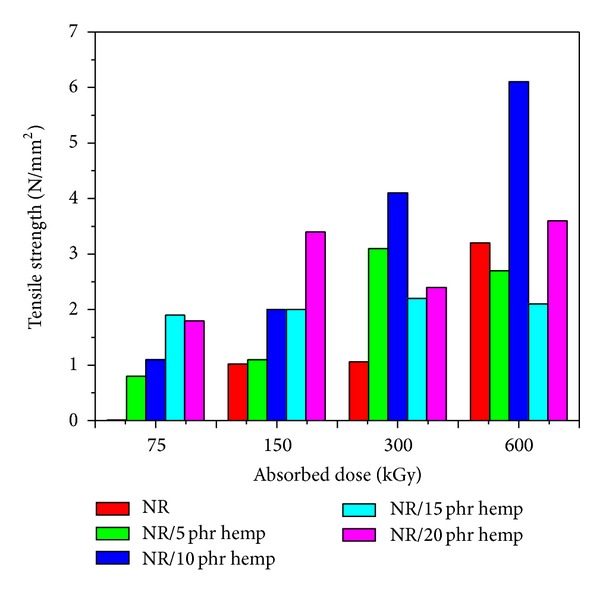
Tensile strength variation as a function of hemp amount and irradiation dose.

**Figure 5:**
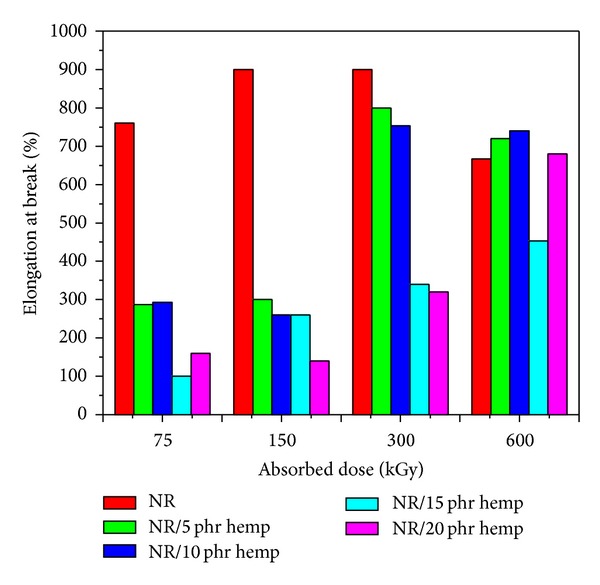
Elongation at break variation as a function of hemp amount and irradiation dose.

**Figure 6:**
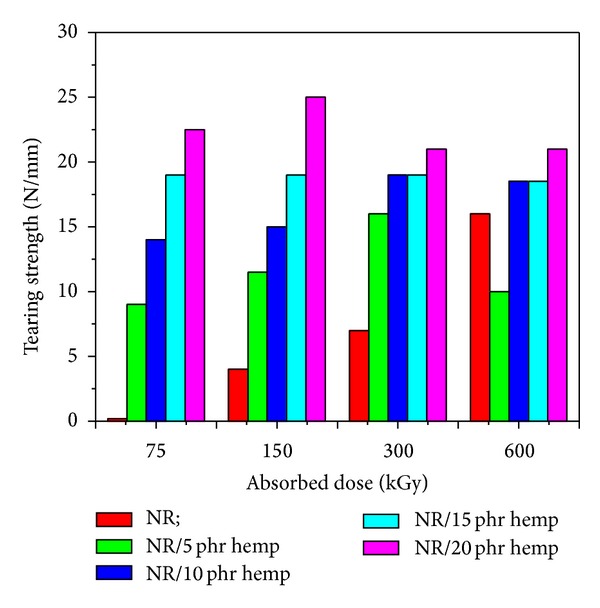
Tearing strength variation as a function of hemp amount and irradiation dose.

Physical and mechanical characteristics of NR/hemp polymer composites crosslinked by electron beam irradiation are presented in Figures 1–6.

The *hardness* (Figure 1) increases with the increase of the absorbed dose and with the fiber amount in polymeric composites. Hardness increases with the increasing of absorbed dose as a result of crosslink density and increases with the hemp amount in polymeric composites because the hemp leads to reinforcement of samples. The maximum value of 70°ShA was obtained at an absorbed dose of 150 kGy and 20 phr hemp amount, much higher than the same samples without hemp (12°ShA). This is because the incorporation of hemp into natural rubber reduces elasticity of the rubber chains, leading to more rigid rubber vulcanizates. *Elasticity* (Figure 2) slightly decreases with the increase of EB dose and varies irregularly when the hemp amount increases.

In the same way, *modulus at 100% elongation* (Figure 3) and tensile strength (Figure 4) increase when the absorbed dose increases and when introducing hemp in natural rubber blends. The maximum value of 3.4 N/mm^2^ (for modulus at 100% elongation) was obtained at an absorbed dose of 150 kGy and 20 phr hemp amount, much higher than the same samples without hemp and vulcanized at the same absorbed dose (0.02 N/mm^2^). In the case of *tensile strength*, the maximum value (4.1 N/mm^2^) was obtained at an absorbed dose of 300 kGy and 10 phr hemp amount, compared to the samples without hemp and vulcanized at the same absorbed dose (1.06 N/mm^2^). The tensile strength of a polymer is a function of crosslink density and energy dissipation. The tensile strength increases with crosslink at lower crosslink density. However, at higher crosslink density the network is so dense that there is little energy dissipation in the matrix and the energy supplied is used for breaking the bonds. At higher crosslink density, the segments of macromolecules become immobile, the system becomes stiffer, and the elasticity decreases.

*Elongation at break *changes (Figure 5) depend also on absorbed dose and fiber amount. *Elongation at break *decreases with the increasing of absorbed dose (compared with the samples without hemp) up to 150 kGy and after that begins to grow. This decrease indicates that the network structure of the crosslinked rubbers becomes tighter and less flexible so that molecular movements are restricted. It can be observed that this parameter (*elongation at break*) decreases with the hemp amount increasing at the same absorbed dose. Obtained values are better compared to those of blends without hemp and vulcanized at the same absorbed dose.

Figure 6 shows that the tearing strength increases when the absorbed dose increases and when introducing hemp in natural rubber blends. The maximum value of 25 N/mm was obtained at an absorbed dose of 150 kGy and 20 phr hemp amount, much higher than the same samples without hemp and vulcanized at the same absorbed dose (7 N/mm). This indicates a vulcanization process.

### 3.3. Gel Content and Crosslink Density of the Blends

Table 1 shows the gel content (mass fraction of the network material resulting from a network-forming polymerization or crosslinking process; the gel fraction comprises a single molecule spanning the entire volume of the material sample), the volume fractions of polymer in the swollen mass (*ν*_2*m*_), and crosslink density (number of crosslinks per unit volume in a polymer network) of the samples vulcanized by electron beam as a function of the absorbed dose and flax content. The determination is based on the absorption of a proper solvent and subsequent swelling of the rubber [33, 34].

The results presented in Table 1 show that when the EB dose and hemp amount increase, there is an increasing in gel content (*G*%), volume fractions of polymer (*ν*_2*m*_), and crosslink density (*ν*) of samples. This is due to the formation of a three-dimensional network structure [35].

### 3.4. Water Uptake

**Figure 7:**
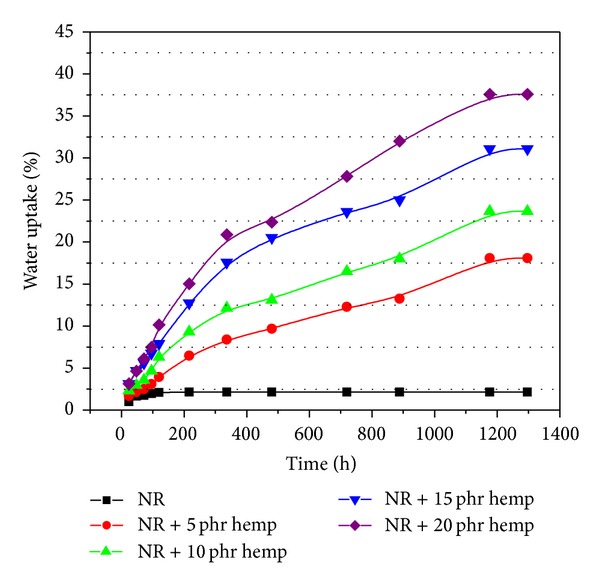
Water uptake of polymeric composites at absorbed dose of 75 kGy.

**Figure 8:**
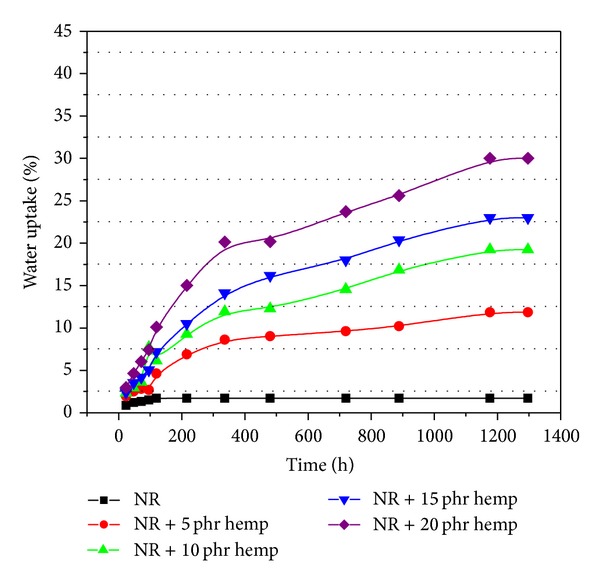
Water uptake of polymeric composites at absorbed dose of 150 kGy.

**Figure 9:**
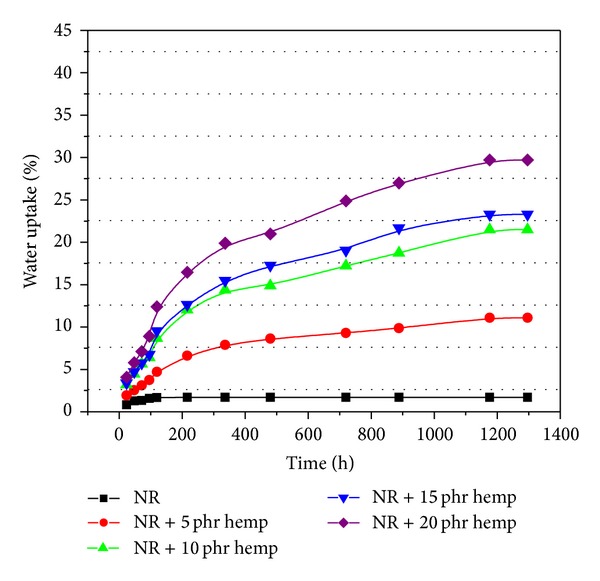
Water uptake of polymeric composites at absorbed dose of 300 kGy.

**Figure 10:**
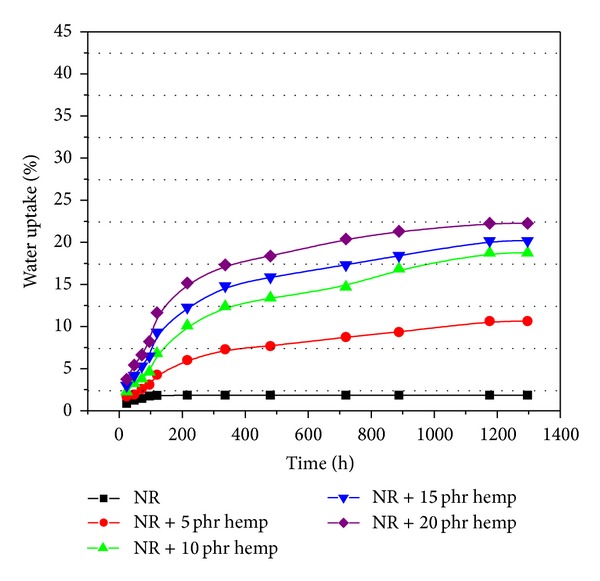
Water uptake of polymeric composites at absorbed dose of 600 kGy.

The water uptake results of samples crosslinked by electron beam irradiation (with and without hemp) are presented in Figures 7, 8, 9, and 10. From these figures it can be observed that the percentage of water absorption in the polymeric composites NR/hemp depended on two parameters: hemp content and absorbed dose. The water uptake increased with increasing of fiber content and decreased with absorbed dose. The increase of water absorption is due to the hydrophilic nature of fiber and the greater interfacial area between the fiber and the elastomer matrix. In polymer composites with fibers, water is absorbed mainly by the fiber because the rubber material is hydrophobic and its water absorbability can be neglected [34].

Irradiation may change the solubility properties of hemp. Activation of the samples by low-dose irradiation (Figures 7–10) is most likely achieved in terms of increased accessibility for the solvent and weakened hydrogen bond networks that translate into better solubility. At higher irradiation dose this effect is suppressed by cross-linking (intra- and intermolecular) [29, 36]. The mechanism of this irradiation activation must again be assumed to be the weakening of the hydrogen bond network, in which hydroxyl groups (H-donating and H-accepting) are converted into carbonyls (only H-accepting) [29, 37].

### 3.5. FTIR Study

**Figure 11:**
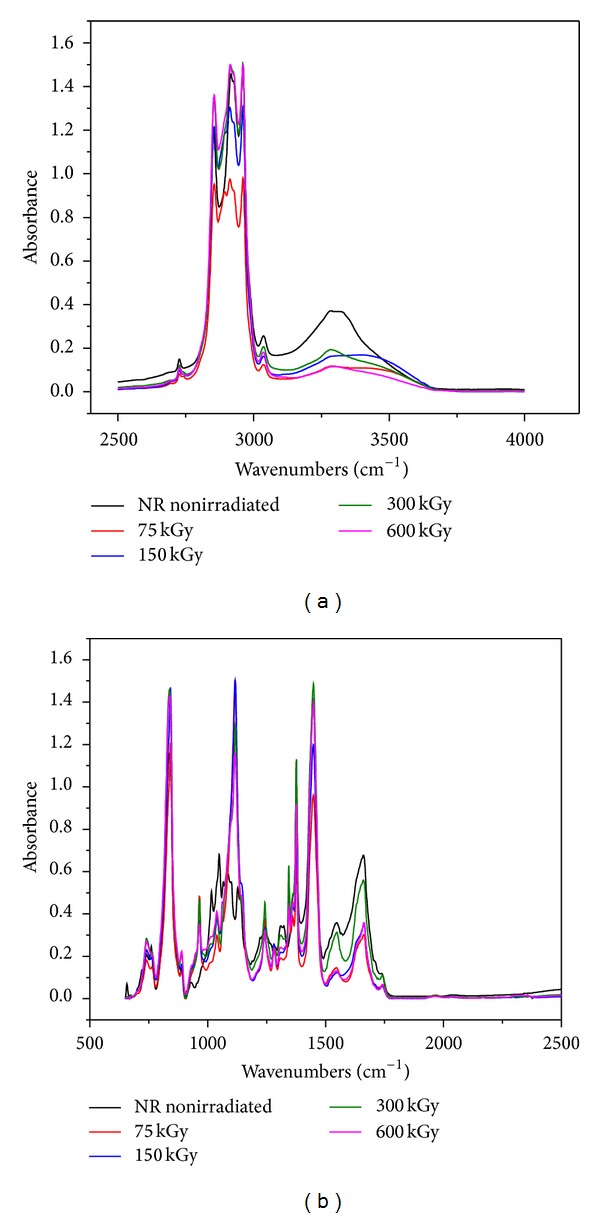
FTIR spectra for NR/hemp mixtures with 5 phr hemp content: (a) between 2500–4000 and (b) between 500–2500.

**Figure 12:**
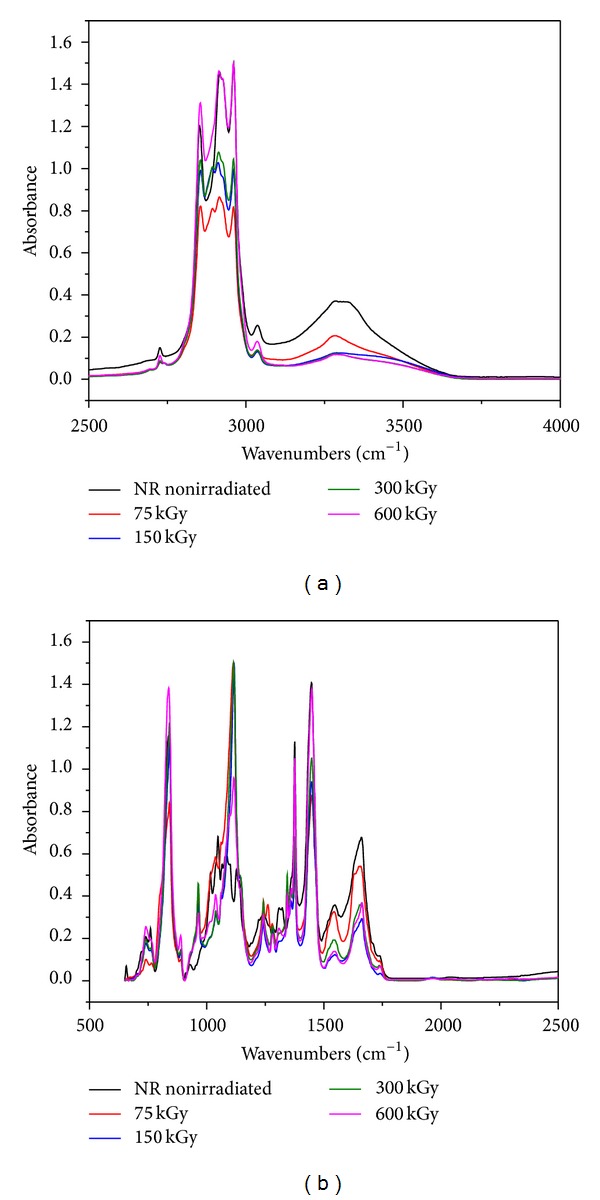
FTIR spectra for NR/hemp mixtures with 10 phr hemp content: (a) between 2500–4000 and (b) between 500–2500.

**Figure 13:**
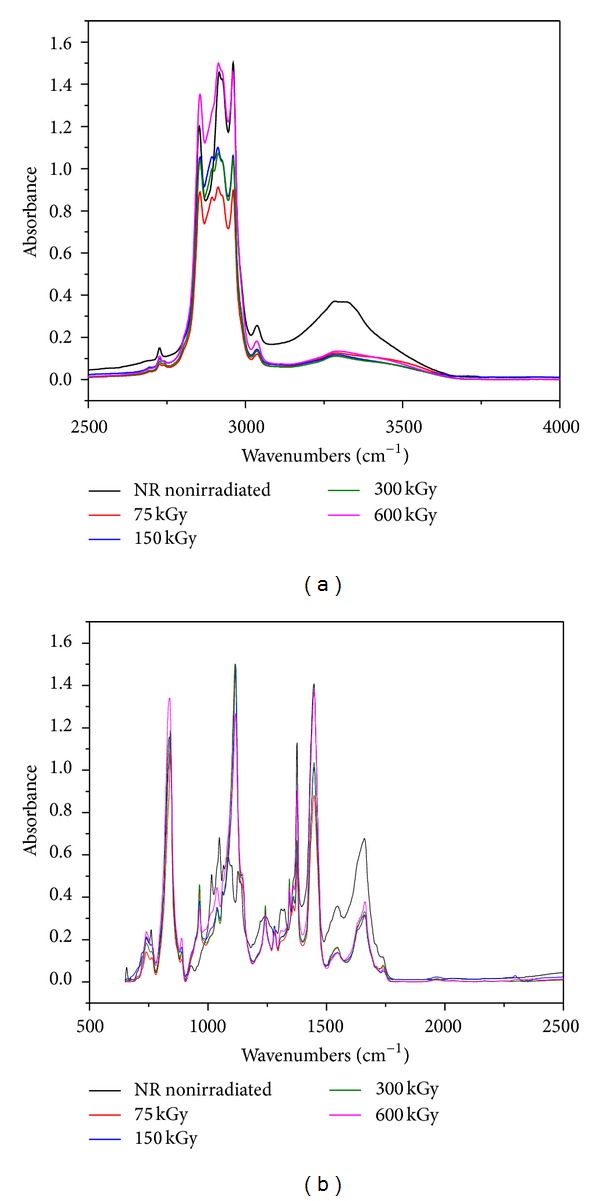
FTIR spectra for NR/hemp mixtures with 15 phr hemp content: (a) between 2500–4000 and (b) between 500–2500.

**Figure 14:**
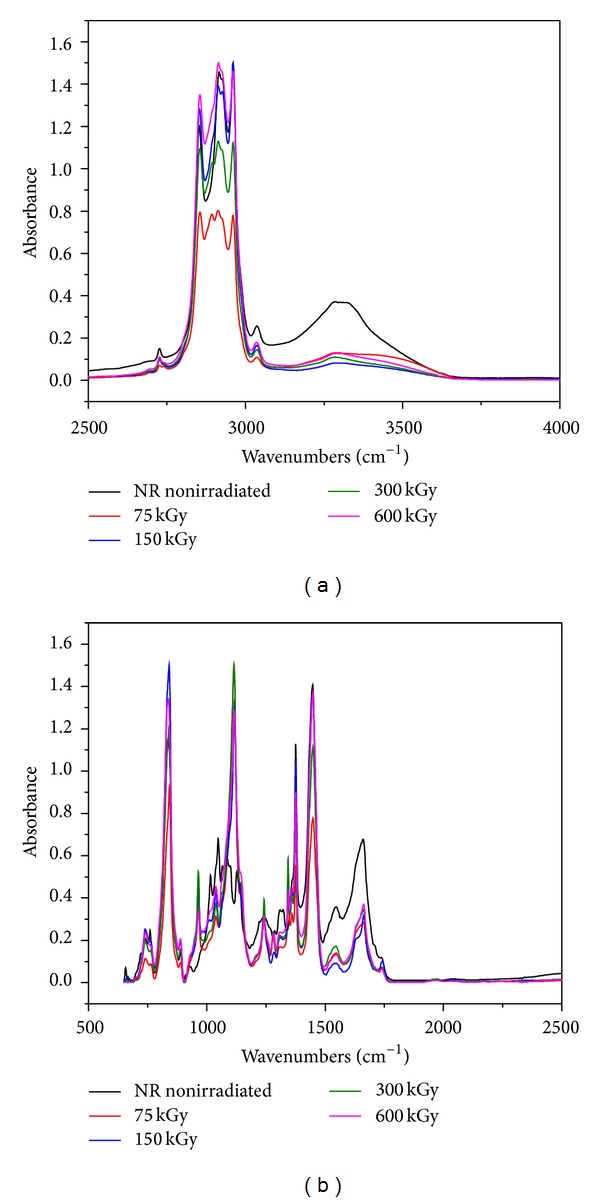
FTIR spectra for NR/hemp mixtures with 20 phr hemp content: (a) between 2500–4000 and (b) between 500–2500.

The main components of our polymer composites are NR and hemp. Natural rubber is composed of hydrocarbons (89.3~92.4 wt%), protein (2.5~3.5 wt%), and other ingredients (4.1~8.2 wt%). The main component of NR is cis-1, 4-polyisoprene with a high degree of long chain branching generally associated with the presence of nonhydrocarbon groups distributed along the chains. Hemp fibers are about 70% cellulose and contain low levels of lignin (around 8–10%), hemicelluloses, lignin, waxes, and so forth. Figures 11, 12, 13, and 14 show the *infrared *spectra and characteristic infrared bands (observed in the region of 4000–560 cm^−1^) of natural rubber with and without hemp, before and after irradiation at absorbed doses of 75 kGy, 150 kGy, 300 kGy, and 600 kGy.

It can be noticed the presence of absorption bands in the spectral region located between 1670 and 1640 cm^−1^, due to the valence vibration of homogeneous double bonds (*ν*_C=C_) in the NR structure. Their intensity decreases for irradiated samples compared with nonirradiated samples. The spectrum exhibits, for nonirradiated NR samples, absorption bands with maxima at 3050–3010 cm^−1^ corresponding to CH stretching in the –CH=CH_2_ group. Irradiation of the polymeric compositions under study between 75 and 600 kGy results in consumption of the double bonds in NR, so that the intensities of these absorption bands decrease and move to the same extent. The specific absorption bands of single bonds corresponding to R_2_C=CH–R group are observed at 850–830 cm^−1^ (see fingerprint region). These changes occur as a result of elastomer crosslinking and double bonds consuming or polymers degradation with the formation of double bonds. The characteristic bands of the saturated aliphatic sp^3^ C–H bonds are observed at 2970–2830 cm^−1^ which are assigned to *ν*_as_ (CH_3_), *ν*_as_ (CH_2_), and *ν*_s_ (CH_2_), respectively (as three corresponding bends) [38]. These bands are specific to natural rubber and cellulose, lignin or hemicellulose, from the hemp fibers existing in the mixture [39]. It can be noticed that with the hemp amount increasing in the mixture, the intensity bands vary out of uniformity. The absorption band of CH_2_ deformation occurs at 1440–1460 cm^−1^ and of CH_3_ asymmetric stretching at 1350–1380 cm^−1^. It is known that the NR contains also other compounds, such as lipids, neutral glycolipids and phospholipids, and so forth. The absorption bands at 3250–3300 cm^−1^ were identified in the proteins and both monopeptides and dipeptides present in natural rubber [40]. This band is specific also for cellulose, lignin and hemicellulose from the hemp fibers existing into the mixture [39]. Band intensity significantly decreases for irradiated samples with the amount of fiber hemp increasing in the mixture. These are the consequences of proteins and peptides degradation. Saeman et al. noted a considerable introduction of oxidized groups upon irradiation of cellulose while making an effort to quantify the amount of introduced carboxylic acid groups [41]. Some authors also observed an increase in carbonyl group content [42, 43]. This effect is observed also for NR/hemp polymer composites irradiated with EB and is highlighted by the presence of the specific C=O bands between 1800 and 1650 cm^−1^. But in our study, hemp fibers, which contain high levels of cellulose, are in the form of filler in an NR polymer matrix. As a consequence, atmospheric oxygen affects these types of irradiated fibers less than in the case of the noticed studies. Although the samples were wrapped in PE foil and after that irradiated in atmospheric conditions, surface degradation of NR/hemp samples can occur. Also, the mechanism of irradiation activation must again be assumed to be the weakening of the hydrogen bond network, in which hydroxyl groups are converted into carbonyls [29]. It can be noticed that with the hemp fiber amount increasing, there is a decreasing of absorption bends intensity in this region, indicating a decrease in the number of double bonds that form with the EB dose increasing (i.e., the number of –OH groups which converted into –COOH decreases). The absorption band around 1730 cm^−1^ was identified to the fatty acid ester groups din NR [44]. In the fingerprint region there are some specific single bends for cellulose, lignin, and hemicellulose from hemp fibers but also for NR; some of them are mentioned above. With the hemp fiber amount increasing, significant changes occur in the specific absorption bands of hemp fiber fingerprint.

## 4. Conclusions

For obtaining new green composites based on natural rubber, active fillers of carbon black or silica type were replaced with hemp fiber, and crosslinking classic system based on sulfur and vulcanization accelerators has been replaced by an ecologic method of crosslinking, namely electron beam irradiation. Our experiments showed that the hemp fibers have a reinforcing effect on natural rubber similar to mineral fillers (chalk, carbon black, silica). Thus, by increasing the hemp amount in the mixtures there occurs an increase in hardness, tearing strength, and crosslinking density and a decrease in elongation at break. When the EB dose increases, is obtained an increase of gel content (*G*%), volume fractions of polymer (*ν*_2*m*_) and crosslink density (*ν*) of samples, due to the formation of a three-dimensional network structure similar to elastomer crosslinking by other crosslinking systems (i.e., sulfur and crosslinking agents). The water uptake increases with fibers content increasing and decrease with absorbed dose. The increasing in water absorption is due to the hydrophilic nature of fibers and activation of the samples by low-dosage irradiation (this leads to an increased accessibility of solvent and weakened hydrogen bond networks that translate into better solubility). At higher irradiation dose this effect is suppressed by crosslinking (intra- and intermolecular), so the water uptake decreases for higher irradiation dose.

## Figures and Tables

**Table 1:** Gel content (*G*%), volume fractions of polymer (*ν*_2*m*_), and crosslink density (*ν*) of samples.

Sample	*G* %	*ν*_2*m*_	*ν* (×10^−4^ mol/cm^3^)
NR 0, 75 kGy	36.24	0.0335	0.0403
NR 0, 150 kGy	93.64	0.0877	0.2476
NR 0, 300 kGy	94.14	0.1164	0.4471
NR 0, 600 kGy	95.92	0.2979	1.1076
NR + 5 phr hemp, 75 kGy	88.40	0.0518	0.0898
NR + 5 phr hemp, 150 kGy	94.44	0.0903	0.2643
NR + 5 phr hemp, 300 kGy	95.55	0.1291	0.5459
NR + 5 phr hemp, 600 kGy	95.96	0.1701	0.9990
NR + 10 phr hemp, 75 kGy	83.70	0.0601	0.1189
NR + 10 phr hemp, 150 kGy	92.99	0.0952	0.2916
NR + 10 phr hemp, 300 kGy	95.32	0.1249	0.5098
NR + 10 phr hemp, 600 kGy	96.37	0.1672	0.9567
NR + 15 phr hemp, 75 kGy	80.29	0.0532	0.0950
NR + 15 phr hemp, 150 kGy	92.91	0.1042	0.3504
NR + 15 phr hemp, 300 kGy	95.55	0.1315	0.5692
NR + 15 phr hemp, 600 kGy	96.53	0.1863	1.2411
NR + 20 phr hemp, 75 kGy	80.30	0.0611	0.1243
NR + 20 phr hemp, 150 kGy	91.67	0.1072	0.3727
NR + 20 phr hemp, 300 kGy	95.71	0.1494	0.7459
NR + 20 phr hemp, 600 kGy	97.20	0.2128	1.6544
